# Supplementary material for: Access to Water and Sanitation Infrastructures for Primary Schoolchildren in the South-Central Part of Côte d’Ivoire
Source: Int J Environ Res Public Health. 2021 Aug 23;18(16):8863. doi: 10.3390/ijerph18168863 (PMC8392625; doi:10.3390/ijerph18168863)
Supplement: Supplementary file 1 [file ijerph-18-08863-s001.zip › ijerph-1321425-supplementary.pdf]

BASELINE SURVEYS IN TAABO –SUDAC/COFER WASH/CSRS PROJECT

Titre : Water, sanitation and hygiene in primary schools in Taabo, Côte d'Ivoire : Assessment of infrastructures, knowledge and practices of management

**1. Questionnaire Information (to be filled by the surveyor)**		
1.1	Survey conducted by *:*	Name : ___________________________
1.2	Survey start time :	
1.3	Date of today :	___ /___ /______<br>Day Month Year
1.4	*Insert codes-ID of the respondent*<br>Site<br>Individu(e)	Code - ID: [ ] [ ]<br>Site Individal
1.5	Did the child give consent ?	□ Yes □<br>□ No □ → ***End of the survey***
1.6	If the interview is not possible, specify the reason	___________________________

**2. Demographic issues**		
2.1	Educational level: What grade/year?	Classe : ___________________________
2.3	Your sex :	□ Male □ Female
2.4	What is your date of birth?	___ /___ /______<br>Day month year<br>□ I don't know.
2.5	How old are you?	Age : _____<br>□ I don't know.

**3. Water, sanitation and hygiene (WASH)**	
**A. PRACTICES / BEHAVIOUR (WASH)**	
4.1	**When do you wash your hands with soap (detergent)?** *(Several answers possible)*<br>□ Befor eating □ Don't wash my hands<br>□ After eating □ I don't know<br>□ After playing □ No response

	□ After defecation<br>□ Other : _______________
4.2	**Pourquoi tu te laves les mains ?**<br>_____________________________
4.3	**What do you usually use to wash your hands?** *(Several answers possible)*<br>□ Water □ Autre : _______________________<br>□ The ash □ I don't know<br>□ Mud □ No response<br>□ Water with soap
4.4	**When should you wash your hands with soap (detergent)?** *(Several answers possible)*<br>□ Before eating □ Don't wash my hands<br>□ After eating □ I don't know<br>□ After playing □ No response<br>□ After defecation<br>□ Other : _______________
4.5	**Do you drink the water provided at school?**<br>□ Yes, drilling □ No<br>□ Yes, from the filter □ Other : _______________________<br>□ Yes, from the well □ I don't know<br>□ Yes, from the tap □ No response
4.6	**Do you bring drinking water from home to school?**<br>□ Yes □ Other : _______________________<br>□ No □ I don't know<br>□ No response
4.7	**The drinking water you brought today, where does it come from?**<br>□ Not brought drinking water today<br>□ From home<br>□ At school<br>□ Other : ______________________________
4.8	**Do you usually ... in a river, a marsh, a lake?** 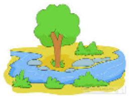<br>**… play ?** □ Yes □ No □ No response<br>**… fish ?** □ Yes □ No □ No response<br>**… do the laundry?** □ Yes □ No □ No response<br>**… work ?** □ Yes □ No □ No response
4.9	Are there latrines at the school **?**<br>□ Yes □ I don't know<br>□ No→ ***continue to 4.13*** □ No response
4.10a	**Are the latrines always open (not closed)?**<br>□ Yes □ I don't know<br>□ No □ No response
4.10b	**Are the latrines clean / well cleaned?**<br>□ Yes □ I don't know<br>□ No □ No response
4.11	**Do you use the latrines at school?**<br>□ Yes→ ***continue to 4.14*** □ I don't know<br>□ No □ No response

4.12	**Why don't you use the latrine at school?**<br>*(Several answers possible)*<br>□ Dirty □ Not enough latrine facilities<br>□ No water □ No privacy<br>□ No soap □ They are not clean/dirty<br>□ Smell □ Other : ___________________________<br>□ Does not work □ No response
4.13	**Where do you relieve yourself if you are in class/school?**<br>□ In the bush □ At home<br>□ Behind the latrines □ Other : ___________________________<br>□ At the latrine □ No response
**B.**	**KNOWLEDGE / ATTITUDES WASH**
4.14a	**Can water cause disease?**<br>□ Yes □ I don't know<br>□ No □ No response
4.14b	**If you said yes à 14.4a, what are the water-related diseases?**<br>(*You can write down several answers)*<br>______________________________ ____________________________<br>______________________________ ____________________________<br>______________________________ ____________________________<br>______________________________ ____________________________<br>______________________________ ____________________________
4.16	**Do you think that if you don't wash your hands you might get sick, for example a stomach ache or diarrhoea?**<br>□ Yes □ I don't know<br>□ No □ No response<br>□ Other : ___________________________
